# Replication and drug resistant mutation of HIV-1 subtype B' (Thailand B) variants isolated from HAART treatment individuals in China

**DOI:** 10.1186/1743-422X-6-201

**Published:** 2009-11-18

**Authors:** Jianping Sun, Liying Ma, Xiaoling Yu, Yang Huang, Lin Yuan, Yiming Shao

**Affiliations:** 1 State Key Laboratory for Infection Disease Prevention and Control, National Center for AIDS/STD Control and Prevention (NCAIDS), Chinese Center for Disease Control and Prevention (China-CDC), Beijing 100050, PR China; 2 AIDS Research Center, Institute of Pathogen Biology, Chinese Academy of Medical Sciences and Peking Union Medical College, Beijing 100730, PR China; 3 Southern Medical University, School of Pharmaceutical Sciences, Guangzhou 510515, PR China

## Abstract

**Background:**

Drug resistant HIV-1 variants were emergent more and more in AIDS individuals with highly active antiretroviral therapy (HAART) treatment. Understanding the replication and drug resistant mutation of HIV-1 variants isolated from HAART treatment individuals of China could help to design appropriate therapeutic strategies for these individuals.

**Methods:**

Use GHOST cell lines to analysis the coreceptor usage of HIV-1 variants. Coculture with PBMCs to analysis the replication capacity. Use RT-PCR to analysis the drug resistant mutation of *pol *gene.

**Results:**

13 HIV-1 variants experienced HAART were included in this study. 5 HIV-1 variants used CCR5 coreceptor (R5), while 8 use both CCR5 and CXCR4 coreceptor (R5X4). The replication capacity of R5X4 variants was no difference with R5 variants in vitro without antiretroviral drugs. Compare the drug resistant mutation between first HIV-1 variants and fourth variants; there were 37 drug resistant mutations in first variants and 32 drug resistant mutations in fourth variants. Only 7 drug resistance mutations were lost after coculture for 4 weeks, and 2 drug resistance mutations were emerged.

**Conclusion:**

These data suggested that the drug resistant level could not reduce in vitro in absence of antiretroviral drugs in few weeks. And maybe helpful for these HAART experienced individuals when change antiretroviral drugs.

## Background

HAART therapies have dramatically reduced the mortality rate from human immunodeficiency virus (HIV) in the developed world[1]. Unfortunately, current therapies are not curative, and many treated patients develop resistance to one or more drugs, which is costly and may lead to complete treatment failure and death. The drug resistant HIV-1 variants emergent in HAART treatment individuals are the major obstacle to antiviral therapy and these drug-resistant variants may transmit in newly infected individuals[2,3].

By the end of 2007, it was estimated that there were 700,000 people living with HIV/AIDS in China. Most of these patients are being treated by antiretroviral treatment regimens. The provision of treatment was expanded to 1,190 counties in 31 provinces (autonomous regions and municipalities) at the end of 2007. http://www.chinaids.org.cn/n435777/n443716/appendix/Joint_Assessment_EN.pdf. it have been reported that the drug resistant variants were emergent with the time of treatment, cause of treatment failure [4-6]. Therefore, design a second-line HAART therapy for HAART failure individuals is urgently needed. Unfortunately, there were also few antiretroviral drugs for consideration.

Better understand the biological phenotype of HIV-1 variants from these HAART experienced individuals could help to design appropriate therapeutic strategies for these individuals. Here, we detected the viral replication level and drug resistant mutation of 13 variants isolated from AIDS patients experienced HAART of Henan and Anhui province in China[6].

## Methods

### HIV-1 variants and cells

HIV-1 variants were isolated from HIV-1 infected individuals of Henan and Anhui province, treated time range 6-18 months. those isolate had been identified as drug-resistance using both genotypic assay and phenotypic assay [6]. SF33, dual tropic, use CCR5 and CXCR4 coreceptor for entry cells, as positive control.

GHOST cells were derived from the human osteosarcoma cell line, HOS, stably express CD4 and the chemokine receptor CXCR4 or CCR5. The GHOST parental cell expressing human CD4 was grown in Dulbecco's modified Eagle's medium supplemented with 10% fetal bovine serum, 1% glutamine, 2% penicillin plus streptomycin, Geneticin (500 μg/ml, Gibco, Paisley, Scotland), hygromycin (100 μg/ml, Gibco). GHOST cells expressing one of the coreceptors CCR5 and CXCR4 were cultivated in medium additionally supplemented with puromycin (1 μg/ml; Sigma, Deisenhofen, Germany)[7,8].

Human peripheral blood mononuclear cells (PBMC) were obtained by Ficoll density centrifugation. were grown in an RPMI 1640(Gibco)-based culture medium supplemented with 10% fetal calf serum (HyClone, Logan, Utah), 2 mM L-glutamine(Gibco), penicillin (100 U/ml), streptomycin (100 mg/ml), and 20 U/ml of recombinant interleukin-2.

### Coreceptor usage

Coreceptor usage was determined with the GHOST(3)-CXCR4 and GHOST(3)-CCR5 cell lines[7,8]. Briefly, GHOST cells were seeded in 24-well plates (Corning Incorporated) at 6*10^4^cells/well. On the following day, the medium was removed, infected with virus stocks in the presence of 8 μg/ml DEAE to enhance infection efficiency. Cells were harvested at 48 h after infection, then using flow cytometer (Elite ESP, Beckman Coulter) to analyze the GFP expression. A total of 10,000 to 15,000 events were scored. We expected an approx 10-fold shift in mean GFP fluorescence of infected cells over non-infected[8]. A positive control (SF33) and cell control were included in each experiment to assess inter-assay variation and cell auto fluorescence.

### Assessment of replication capacity

At least 2 HIV-1 negative PBMC and HIV-1 variant were coculture[9]. Briefly, 5*106 PBMC cells from normal seronegative donors were stimulated with 1 ug of PHA per ml for 2-3 days, were infected overnight with 2 ng of p24 antigen equivalent of each viral isolate, washed, and resuspended in complete medium supplemented with recombinant interleukin-2. Aliquots of virus supernatant were collected every 2 to 3 days and monitored for p24 antigen production over 13 days with a commercially available ELISA (Vironostika HIV-1 Microelisa system, bioMérieux, France)

### Nucleic acid extraction, amplification and sequencing

RNA was extracted from the plasmas and HIV variants using a QIAamp^® ^Viral RNA Mini Kit (Qiagen Inc., Chatsworth, CA).

For synthesis of cDNA, 5 μl extracted RNA, 10 pmol downstream PCR primer (RT21: CTGTATTTCTGCTATTAAGTCTTTTGATGGG; HXB2 3509-3539), M-MuLV reverse transcriptase (NEW ENGLAND BioLabs) and Ribonuclease Inhibitor (TaKaRa Biotechnology) were run for 60 min at 45°C in a thermal cycler (GeneAmp^® ^PCR system 9700, Applied Biosystems, Foster City, CA). A nested PCR strategy was employed to amplify the 1,100-bp RT fragment. cDNA (10 μl) was used as a template and the outer primer set (MAW 26: TTggAAATgTggAAAggAAggAC; HXB2 2027-2050 and RT21) was used in the first round of PCR. The amplification was done at 94°C for 5 min, followed by 35 cycles at 94°C for 20 sec, 55°C for 20 sec and 72°C for 2 min, and finally an extension of 7 min at 72°C. The first-round PCR product (5 μl) and the inner primer set (PRO-1: CAgAgCCAACAgCCCCACCA; HXB2 2147-2166 and RT20: CTgCCAgTTCTAg CTCTgCTTC; HXB2 3441-3462) were used in the second round of PCR. The amplification was performed at 94°C for 5 min, followed by 35 cycles at 94°C for 20 sec, 55°C for 20 sec, and 72°C for 1 min, and finally an extension of 7 min at 72°C.

The nested PCR product was purified using a QIAquick Gel Extraction Kit (Qiagen Inc., Chatsworth, CA). DNA was sequenced with the ABI 3100 DNA Sequencer (Applied Biosystems Inc). The primers used for DNA sequencing were PRO-1, RT-20, RT-A (gTTgACTCAgATTggTTgCAC; HXB2 2519-2539), and RT-B (CCTAgTATAAACAATgAgACAC; HXB2 2946-2967).

The pol gene of HIV-1 subtype B' were sequenced and compared to the consensus B reference sequence, using the HIVdb software (Stanford HIV Drug Resistance Database, http://hivdb.stanford.edu) to detect drug resistance mutations.

### Statistical Analysis

Difference between R5 drug resistance variants and R5X4 drug resistance variants were compared using T analysis. Two-tailed P values less than 0.05 was considered statistically significant. All statistical analyses were completed by the GraphPad5.0 software.

## Results

### Characterization of HIV-1 variants

13 HIV-1 variants were isolated from AIDS patients (4 male and 9 female; average 40.3 years old) were recruited for this study. 4 patients received AZT + ddI + NVP, and 9 were treated with d4T + ddI+ NVP for 6 months or longer. The average viral load was 8.08 × 10^5 ^copies/ml (range from 5.0 × 10^2 ^~ 4.0 × 10^6 ^copies/ml), and the average CD4 count was 151 cells/ul (range from 63 ~ 348 cells/ul).

Using GHOST cell line to determined the coreceptor usage, We found 5/13 viral variants were CCR5-tropic(R5) as determined by parallel infection of GHOST(3)-CXCR4 and GHOST(3)-CCR5 cells, 8/13 viral variants were CCR5 and CXCR4-tropic(R5X4). Among 13 drug-resistant variants, the CD4 T counts of R5X4 variants were 97.9 (range from 63 to 186), while that of R5 variants were 236.2 (range from 144 to 348/μl; *P *= 0.04). The R5X4 variants mainly appeared in CD4 counts <200/ul. Viral load of R5X4 variants were 4.91 ± 1.58, while that of R5 variants were 5.67 ± 0.33. There was no difference in viral load between R5X4 variants and R5 variants (*P *= 0.32). The average time under HAART were no difference between R5X4 variants and R5 variants, 13.1 and 15.6 months, respectively (*P *= 0.4).

### Replication of HIV-1 variants in stimulated PBMCs without antiretroviral drugs

**Figure 1:**
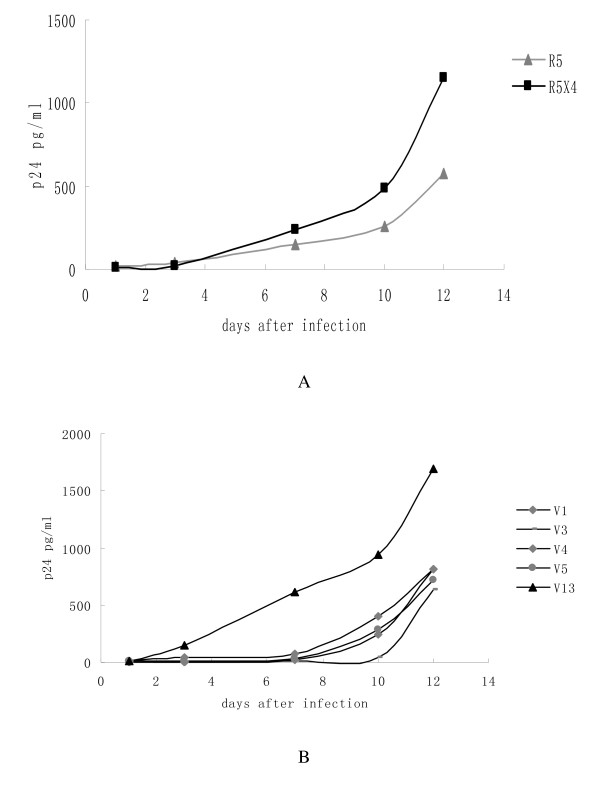
**Replication kinetics of HIV strains isolated from patient's PBMCs**. A: compare the mean p24 antigen level of R5 variants and R5X4 variants; B: p24 level of one R5 variants and 4 R5X4 variants. Virus replication was monitored by measuring p24 antigen level in supernatant of duplicate cultures of each virus.

HIV-1 variants growth kinetics analyzed by determined the level of p24 antigen at 1, 3, 7, 10, 12 days after HIV-1 variants infection. All HIV-1 variants were able to yield productive infections in PBMCs assay. But all HIV-1 variants produce less 10000 pg/ml at 12 day. Compare the p24 values at 1, 3, 7, 10, 12 day, the growth kinetics of R5-tropic HIV-1 variants was similar with R5X4-tropic HIV-1 variants (fig 1A, p > 0.05).

Although the mean p24 antigen level of R5-tropic variants was lower than that of R5X4 variants. In some case, R5 variants replicated more efficiently than R5X4 viruses, for example, V13 variant was more efficiently than V1, V3, V4 and V5 variant (fig 1B).

### Drug-resistant mutation between first and fourth HIV-1 variants

**Figure 2:**
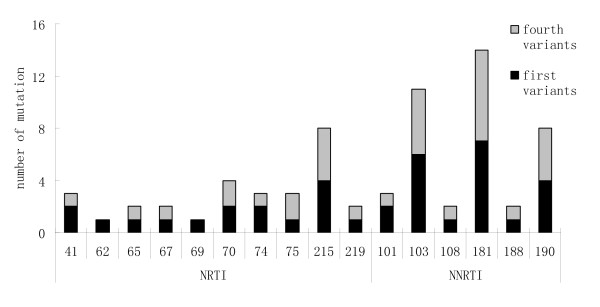
**Prevalence of first and forth HIV-1 variants with mutations for genotypic resistance to NRTI and NNRTI**.

HIV-1 variants placed in a humidified chamber at 37°C with 5% CO_2 _air and maintained for 4 weeks without antiretroviral drugs. Compare the drug resistant mutations of *pol *gene between first and fourth variants. There was no primary drug mutation of protease both in first and fourth HIV-1 variants. In first HIV-1 variants, 37 primary drug mutations including 16 NRTI mutations and 21 NNRTI mutations were detected. For NRTI, there were 4 HIV-1 variants were mutated at 215 site, including T215Y and T215F mutation. The most frequency mutations of NNRTI were at 181 and 103 site (fig 2). 32 primary drug mutations including 13 NRTI mutations and 19 NNRTI mutations were detected in fourth HIV-1 variants. The most frequency mutation of NRTI was at 215 site. There were 7 mutations at 181 site, and 6 mutations at 103 site, for NNRTI.

Compare the drug resistant mutation at 215 site between first and fourth variants, there were no difference. The mutation at 181 site was lost in one HIV-1 variant, and emergent in another HIV-1 variant. K103N mutation was lost in one variant after coculture.

The NRTI drug resistant mutations lost in 3 strains after coculture, including T69APST, M41LM, and A62V. One drug resistant mutation (L74V) changed to V75T. The NNRTI drug resistant mutations lost in 3 strains, including K103N, K101*EKQ, Y181C and V108FILV. 2 drug resistant mutations (V108I and Y181C) emergent after coculture. One drug resistant mutation (G190EV) changed to G190A (table 1).

**Table 1:** drug resistant mutation sites of primary variants and variants after culture 4 weeks

		first variants		fourth variants	
			
Virus variants	tropism	NRTIs	NNRTIs	NRTIs	NNRTIs
V1	R5X4	-	**K103N**	-	-
V2	R5	-	K101*EKQ, Y181C, G190A	-	G190A
V3	R5X4	T215Y	K103N, Y181C	T215Y	K103N, Y181C
V4	R5X4	D67N, K70R, T215F, K219Q	K103N, Y181C	D67N, K70R, T215F, K219Q	K103N, Y181C
V5	R5X4	**L74V**	Y181C	**V75T**	***V108I***, Y181C
V6	R5X4	-	K103N, Y181C	-	K103N, Y181C
V7	R5X4	M41L, L74V, T215F	K101E, Y181C, G190S	M41L, L74V, T215F	K101E, Y181C, G190S
V8	R5	K65R	K103N, Y181C	K65R	K103N, Y181C
V9	R5	**T69APST**	**V108FILV**	-	-
V10	R5	**M41LM**, T215Y	K103N, G190A	T215Y	K103N, G190A
V11	R5X4	**A62V**, K70KN, V75T	Y188L	K70N, V75T	Y188L
V12	R5X4	-	**G190EV**	-	**G190A**
V13	R5	-	-	-	***Y181C***

-: none; **bold**: lost after culture; **bold **and *italic*: emergent after culture; **bold **and underline: changed after culture.

## Discussion

HIV-1 entry into cells requires interactions with certain coreceptors in addition to CD4. Several studies have shown that the CCR5 acts as the major coreceptor for primary non-T-cell-line-adapted viruses[10,11]. Virus variants use CCR5 co-receptor for entry was R5 phenotype, also referred to as non-syncytium-inducing (NSI) viruses. X4 phenotype referred to SI phenotype and T-cell-line-adapted HIV-1 strains instead use the CXCR4 as coreceptor[12,13]. The coreceptor antagonist such as Maraviroc, is a new class of drugs approved to apply in combination with other antiretroviral drugs for the treatment of adults with CCR5-tropic HIV-1, who have been treated with other HIV medications and who have evidence of elevated levels of HIV in their blood (viral load)[14]. We determined the coreceptor usage of 13 drug resistant variants based the GHOST cell line. Interesting, there were 8(61.5%) drug resistant variants were use CCR5 and CXCR4 coreceptor for entry cells (R5X4). In our previous work, we found that 40%-50% patients with HIV-1 subtype B' infection native to HAART treatment, would switch from R5 phenotype to R5X4 phenotype with disease progression[15]. Therefore, the R5X4 phenotype would more general in drug resistant variants from HAART treatment individuals, suggested that the inhibitor of CCR5 may be less useful in these individuals.

CD4 T cell counts of all R5X4 phenotype were less than 200 cells/ul (mean is 97.9 ± 50.2), while that of R5 phenotype range from 144 to 348 cells/ul (mean is 236.2 ± 91.6, *P *= 0.04). There was no difference between R5X4 phenotype viral load (4.91 ± 1.58) and R5 phenotype viral load (5.67 ± 0.33, *P *= 0.32). It have been proved that the emergence of SI viruses is associated with an accelerated decrease in CD4 T cell count, rapid disease progression and the establishment of AIDS[16,17]. In HAART treatment individuals with HIV-1 subtype B' infected, the emergence of variants use CXCR4 coreceptor may be imply the severe disease progression.

It is believed that replication capacity is an important determinant of HIV-1 pathogenicity and transmissibility. We analysis the replication properties of 13 drug-resistant variants using PBMC assay. The data show all drug resistant variants were able to yield productive infections in PBMCs. Suggested that drug-resistant HIV-1 isolates can bear adaptive mutations that allow for wild-type-level replicative function, thereby overcoming the potential defects associated with the genetic changes necessary for drug resistance. Interestingly, the p24 antigen level of drug-resistant variants were lower than 10 ng at 7 day after infection, while the drug sensitivity variants usually reach 10 ng at 7 day after infection in our lab previous work (data unpublished). This result was coincides with several other reports [18-20], that the drug resistant HIV variants showed a decreased replication capacity than wild-type strains in the absence of antiretroviral therapy.

The drug resistance mutation in protease (PR) and reverse transcriptase (RT) genes responsible for escape drug inhibition and some may responsible for moderate viral fitness[21]. We compared the drug resistance mutation between first variants and fourth variants in the absence of drugs. Only 7 out of 37 mutations were lost and emerged 2 mutations compare the first and fourth variants. Interestingly, the mainly primary mutations such as T215F, Y181C were stable in vitro environment in the absence of antiretroviral drugs. These data suggested that the drug resistant variants were stable in vitro culture in the absence of drugs. Simon[18] also show that in vitro culture did not reduce the level of drug resistance. Therefore, when design the second-line HAART therapy for HAART failure individuals, it should substitute the drugs belong to new class such as fusion inhibitors or integrase inhibitors, instead of the same class.

Due to the decreased replication capacity of drug resistant variants, there is a hypothesis that when antiretroviral treatment is interrupted or terminated, drug-resistant variants in the quasispecies are rapidly replaced by the most fit wild-type virus. And it has been noted that after cessation of treatment following failure, resistant virus is often replaced by wild-type virus[22]. These have clinical relevance in terms of selecting optimal therapies and reducing the rate of progression, as well as on the modeling of the epidemiology of transmission of resistant HIV-1strains. However, the drug resistant mutations in vitro in absence of drugs were persistent and only 7 mutations were lost after passage suggested that the drug resistant variants were predominant in culture. On the other hand, reversion to the wild type amino acid appears to occur infrequently[23]. Our data also show that the drug resistant mutation to wild type can occur at 41, 62, 69, 101, 103, 108, 181, ect. Therefore, the cessation of treatment should be carefully and discussed more[24]. Furthermore, even less fit, drug resistant variants will probe the sequence space and fitness landscape under drug pressure and eventually evolve as a more fit isolate, i.e. one that is stable in the quasispecies upon removal of drug pressure and that can be transmitted to new human recipients[25]. Here, we use HIV-1 clinical variants instead of recombinant infectious clones to measure viral biological phenotype, and should be noted that our drug resistant variants were HIV-1 quasispecies, since the quaisispace variants responsible for the whole viral replicative capacity under the microenvironment.

## Competing interests

The authors declare that they have no competing interests.

## Authors' contributions

JS, XY, YH performed experiments; JS, LM, and LY analyzed data and prepare figure, LM, and YS designed the research.
